# Supplementary material for: Development and Validation of a Questionnaire to Assess Adherence to the Healthy Food Pyramid in Spanish Adults
Source: Nutrients. 2020 Jun 3;12(6):1656. doi: 10.3390/nu12061656 (PMC7352209; doi:10.3390/nu12061656)
Supplement: Supplementary file 1 [file nutrients-12-01656-s001.pdf]

Supplementary A: Adherence to Pyramid Questionnaire (APQ)

Question 1 (Q1). Do you exercise? ☐ No/☐ Yes (cross each day that you do the sport activity)

	Intensity	Duration	Sun	Mon	Tue	Wed	Thu	Fri	Sat
**Aerobic**	☐ Light<br>☐Moderate<br>☐High	_____min/session							
**Strength**	☐ Light<br>☐Moderate<br>☐High	_____min/session							

Question 2 (Q2). Current employment situation:

☐ Studying.
☐ Unemployed/retired/sick since ______ months and used to work as a _______
☐ Working as a (profession) ____________

Question 3 (Q3). Complete the following sentence:

At work, I am ____ hours seated, and when I get home I am ___ hours seated. During my free time, I enjoy (different activities described in Q1) _____ and I usually do them____ times/week.

Question 4 (Q4). Tobacco.

☐ I have never smoked.
☐ I don't smoke since ____________ months.
☐ I don't smoke, but I'm in smoking environments.
☐ I smoke ________ cigarettes/day for ____________ years.

Question 5 (Q5). Regular medication: ☐ No/☐ Yes: Which one? : _________________

Question 6 (Q6). Food supplements: ☐ No/☐ Yes: Which one? : __________________

Question 7 (Q7). Do you agree with the following statements? (0 "No"; 3 "Completely").

The weighing scale has great power over me	0 1 2 3
The weighing scale can change my mood	0 1 2 3
On regular basis, I have cravings for specific food	0 1 2 3
I find it hard to stop eating sweet foods, like chocolate	0 1 2 3
I have trouble controlling the amounts of what I eat	0 1 2 3
I eat when I'm stressed, angry or bored	0 1 2 3
I eat more of my favorite foods when I'm alone	0 1 2 3
I feel guilty when I eat food that I know I shouldn't eat	0 1 2 3
When I arrive tired from work, I feel like I lose my diet's control	0 1 2 3
I feel food controls me instead of me controlling her	0 1 2 3

Question 8 (Q8). How satisfied are you with your sleep? (0 "Highly Unsatisfied", 6 "Highly satisfied").

Question 9 (Q9). Total hours of sleeping per day _____

Question 10 (Q10). Time spent on falling asleep (minutes): ☐ 0–15/☐ 16–30/☐ 31–45/☐ 46–60/☐ >60.

Question 11 (Q11). Number of awakenings: ☐ None/☐ 1/☐ 2/☐ 3/☐ 4.

Question 12 (Q12). The meals you eat each day: ☐ Breakfast/☐ Midmorning/☐ Lunch/☐ Snack/☐ Dinner/☐ Other

Question 13 (Q13). How often do you eat fried foods? : ☐ Never/☐ ≥1/month/☐ ≥4/month/☐ ≥2/week/☐Daily

Question 14 (Q14). The culinary techniques: mark 3 that you use most: Raw – Steamed– Microwave – Sauteed – Baked – Stew – Grilled – Fried – Boiling – Charcoal–grilled.

Question 15 (Q15). Glasses of water per day (4 glasses = 1 L) ______

Question 16 (Q16). Cups of tea and/or coffee per day ______

Question 17 (Q17). Usual sweeten: ☐ Sweetener/☐ White or brown sugar/☐ Honey/☐ None/☐ Other: _____ Amount: _____ ☐ Packets/☐ Spoonful/☐ Pills/☐ Drops

Question 18 (Q18). Complete the table regarding the consumption of soft drinks.

Type	No. of cans	Consumption frequency
☐ Sugared<br>☐ Light<br>☐ Zero<br>☐ Energy drinks		☐ Daily<br>☐ Weekends. At least 1/week<br>☐ Occasionally < 1/week

Question 19 (Q 19). Complete the table regarding the consumption of wine and beer.

	Volume	No. Of cups per day
**Wine**	☐ 50 mL<br>☐ 100 mL<br>☐ 200 mL	
**Beer**	☐ 150 mL<br>☐ 250 mL<br>☐ 330 mL	

Question 20 (Q20). Spirit drinks intake: ☐ Never/☐ Occasionally/☐ Weekends/☐ Daily

Question 21 (Q21). How often do you consume cereals? (0 "No"; 3 "Completely").

(0 "Never"; 5 "Daily")		(0 "Daily"; 5 "Never")	
Oats	0 1 2 3 4 5	Commercial cereals	5 4 3 2 1 0
Pasta	0 1 2 3 4 5	Pizza	5 4 3 2 1 0
Rice	0 1 2 3 4 5	White soft bread	5 4 3 2 1 0
Integral soft bread	0 1 2 3 4 5		
Bread	0 1 2 3 4 5		
Quinoa	0 1 2 3 4 5		
Legumes	0 1 2 3 4 5		
Potatoes	0 1 2 3 4 5		
Nuts	0 1 2 3 4 5		
Seeds	0 1 2 3 4 5		

Question 22 (Q22). How much fruit do you consume?

Fruits	Pieces/day
Fruits:	
Juices	Glasses/week
Natural:	
Commercial:	

Question 23 (Q23). How often do you consume vegetables? (0 "Never"; 5 "Daily").

Green leafy vegetables	0 1 2 3 4 5	Corn	0 1 2 3 4 5
Green beans	0 1 2 3 4 5	Pepper	0 1 2 3 4 5
Asparagus	0 1 2 3 4 5	Tomato	0 1 2 3 4 5

Cauliflower/Broccoli	0 1 2 3 4 5	Carrot	0 1 2 3 4 5
Artichokes/Brussels sprouts	0 1 2 3 4 5	Garlic/Onion	0 1 2 3 4 5
Zucchini/Eggplant/Pumpkin	0 1 2 3 4 5	Mushrooms	0 1 2 3 4 5

Question 24 (Q24). What type of fat do you use for… a) cooking: ___ b) Salads: ___ c) Toasts: ____ d) Between butter and margarine, you prefer: ___

Question 25 (Q25). How often do you consume dairy products?

	(0 "Never"; 3 "Daily")		(3 "Never"; 0 "Daily")
Semi–skimmed milk	0 0.5 1 2 3	Sweetened yogurt	3 2.5 2 1 0
Skimmed milk	0 0.5 1 2 3	Cured cheese	3 2.5 2 1 0
Natural yogurt	0 0.5 1 2 3	Blue cheese	3 2.5 2 2 1
Skimmed yogurt	0 0.5 1 2 3	Whole milk	0 1 3 2 1
Fresh cheese	0 0.5 1 2 3		

Question 26 (Q26). How often do you consume animal protein?

	(0 "Never"; 3 "Daily")
Eggs	0 1 2 4 3
White meat	0 1 3 4 1
Red meat	3 4 3 0 –1
Processed meat	3 3 2 0 –1
Fish	0 1 3 4 1

Question 27 (Q27). Do you eat seafood at least once a month? ☐ No/☐ Yes.

Question 28 (Q28). How often do you eat "other products"?

	(3 "Never"; –1 "Daily")		(0 "Never"; 1 "Daily")
Soluble cocoa	3 2 1 0 –1	Dark chocolate	0 2 2 1 1
Milk Chocolate	3 2 1 0 –1	Pickles	0 2 2 1 1
White chocolate	3 2 1 0 –1		
Sweets	3 2 1 0 –1		
Pastries	3 2 1 0 –1		
Biscuits	3 2 1 0 –1		
French fries	3 2 1 0 –1		
Fried rashers of bacon	3 2 1 0 –1		
Sauces	3 2 1 0 –1		

Supplementary B: AP–Q Correction

Category 1: Physical Activity

Physical activity can only have values ≥ 0, being 21 the maximum achievable score.

Q1. If the physical exercise response is positive, the scores related to the intensity and duration of each exercise time should be summed and multiplied by the number of days per week. The maximum score is 21 points and is achieved by performing moderate exercise for 60 min or more each day of the week.

Q2. The maximum score (10 points) is obtained if the respondent has a profession that involves physical effort. In the case of being professionally active but being retired/sick for less than 1 month, 5 points are added; whereas, if the job break is greater than 3 months, 2 are added. For professionals with passive jobs or individuals with no job, the score is 0.

Q3. 10 points will be added if the activity performed during free time involves physical exercise and is performed 6 to 7 times a week. This score decreases if the frequency goes down, being 7 points for 4–5 times a week, 4 for 2–3 and 1 point if the activity is performed only once.

The final score for category 1 is obtained by summing the scores of the three questions.

Category 2: Healthy Habits and Culinary Techniques

Dimension I: Lifestyle.

Q4. The maximum score of 5 points is obtained if the respondent has never smoked. If you have been a former smoker for more than 2 years you get 3 points, while if you quitted less than 24 months ago or are a passive smoker, you get no points. In the case of smokers, the tobacco index is carried out (number of cigarettes * years smoking/20) and the scores are always negative (from –1 to –7 points).

Q5. The affirmative answer subtracts 1 point.

Note: future validation of the questionnaire should consider this item non–penalty if there are special diets (vegans), situation (pregnancy, lactation) or diseases, which require supplementation.

Q6. The affirmative answer subtracts 1 point.

The final score for dimension 1 is obtained by summing the scores of the three questions.

Dimension II: Emotional Balance.

Q7. The different statements are evaluated as a Likert scale in which 0 is "No agreement" and 3 is "Very agreement". Since the statements are related to a dietary pattern based on the emotional intake of obese people, the scores range from 3 points for statements marked as "Disagree" and 0 points for those on which respondents strongly agree. The score is obtained by dividing the sum of the answers by 10.

Dimension III: Sleep Hygiene.

Q8. Likert scale in which "Highly unsatisfied" scores 0, and "Highly satisfied" scores 6 points.

Q9. Sleep of 7–8 h has the highest score (3 points), sleep of 5–6 h or 9–10 h (2 points), while sleep of less than 5 or more than 10 h, scores 1.

Q10. The longer latency time, the lower score obtained: 0–15 min (+2 points), 16–30 min (+1 point), 31–45 min (0 points), 46–60 min (–1 point) and over 60 min (–2 points).

Q11. The more awakenings during sleep, the lower the score obtained: no awakening (+2 points), 1 time (+1 point), 2 times (0 points), 3 times (–1 point) and more than 3 times (–2 points).

The final score for dimension III is obtained by summing the scores of the four questions.

Dimension IV: Culinary Techniques.

Q12. One point is added for each meal, except for breakfast, which is worth 2. In case of selecting "other meals" and the sum is greater than 5, subtract 1 point.

Q13. The consumption of fried food is rated negatively from –0.5 if done monthly to –3 points if consumed daily.

Q14. Grilled, fried, stewed and sautéed cooking techniques are rated negatively (–3 and –1 point), while the intake of raw, steamed, grilled, cooked, baked or microwave–cooked foods are rated positively (3, 2 and 1 point).

The final score for dimension IV is obtained by summing the scores of the three questions.

The final score for category 2 is obtained by dividing the sum of the four dimensions by 4.

Category 3: Hydration

Dimension I: Water Intake.

Q15. The number of glasses indicates the obtained score, being 5 the maximum attainable score.

Q16. The number of cups indicates the obtained score divided by 2, with 2.5 being the maximum achievable score.

Q17. The sweetening of drinks is evaluated negatively, with the sweetener being the least negative (–0.5) and sugar or honey obtaining the lowest score (–2). In addition, the amount of spoons or drops used can subtract up to 3 points in the case of using 4 or more tablespoons.

The final score for water dimension is obtained by adding the scores of the three questions.

Dimension II: Soft Drinks.

Q18. Scores should be summed for the number of cans (—0.5 points for 1 can to —4 points if 5 or more are consumed) and the frequency of consumption (—1 if sporadic to —3 if daily). In addition, if the drinks are energetic and/or sugared, 0.2 is subtracted, while 0.1 if they are light. The score for this question can only have negative values.

Dimension III: Wine and Beers.

Q19. The number of drinks per week provides a score ranging from 0 to −1.5 if less than 7 drinks per week or more than 22 respectively are consumed. To this score, the size of the glass should be subtracted, whose values range from 0.2 for the smallest size to —0.8 for the largest. Non–alcoholic beers are considered as water, and its score will be added to the water intake category, with a maximum of 5 points.

Dimension IV: Spirit Drinks.

Q20. Consumption of alcoholic beverages is rated negatively, from –1 if it is sporadically consumed to –6 points if it is daily. It does not score if the individual does not consume alcohol.

The final score for category 3 is obtained by summing the four dimensions considered.

Category 4: Grains, Seeds and Legumes

Q21. Likert scale in which "Never" scores 0 and "Diary" scores 5 points for all items except commercial cereals, pizza and white soft bread, in which case "Never" scores 5 points and "Diary" 0. Items such as industrial pizza, pasta, rice and white bread are multiplied by a 0.5 factor. In addition, 1 point is added if the consumption of whole grains is affirmative.

The final score for category 4 is obtained by dividing the sum of items by 11 and the sum of points for each whole grain.

Category 5: Fruits

Q22. Whole fruit consumption scores from 2 points if a single piece is consumed to 5 points for consumption of 3 to 4. Natural juices score positively, and their maximum score is obtained by consuming 1–2 juices per week, while commercial juices score negatively in all cases, with 5 or more per week being the frequency that obtains the lowest score (–3).

The final score for category 5 is obtained after the summation of fruits, natural and commercial juices consumption, being 5 points the maximum achievable score.

Category 6: Vegetables

Q23. Likert scale in which "Never" scores 0 and "Diary" scores 5 points for all items.

The final score for category 6 is obtained by dividing the sum of points by 12.

Category 7: Oil Type

Q24. In each section it is positively valued (+ 1 point) if the oil used comes from olives and +2 points if it is Extra Virgin. However, if butter or margarine were used, 1 point would be subtracted. In the last section, 1 point is deduced if the answer is "Margarine".

The final score for category 7 is obtained by summing the four sections of question 20.

Category 8: Dairy Products

Q25. Likert scale where "Never" is 0 and "> 1 time/week" is 3 points for semi–skimmed and skimmed milk, natural yogurt and 0% MG, and fresh cheese, while for items such as sugared yogurt, cured cheese and blue, "Never" scores 3 points and "> 1 time/day" scores 0 points. Whole milk is an exception, as the maximum score (+ 3 points) is reached if consumption is 1–3 times/week.

The final score for category 8 is obtained by dividing the sum of points by 3.

Category 9: Animal Proteins

Q26. Likert scale in which "Never" is 0 points and the maximum score (+4 points) is reached if the consumption is 3 to 6 times per week for white meat, fish and eggs. However, red meat achieves the highest score (+ 4 points) if consumed 1 to 2 times per month and processed meat (+3 points) if never consumed.

Q27. If affirmative, subtract 1 point.

The final score for category 9 is obtained by dividing the sum of points from question 22 by 3 and question 23.

Category 10: Snacks

Q28. Likert scale where "Never" is 3 points and "> 1 time a day" is –1 point on all items except dark chocolate and pickles.

The final score for category 10 is obtained by dividing the sum of points by 11.

The final score for each category is divided by the highest possible score for that category, resulting in values ranging from –1 to 1. After summing all categories, a final score of up to 10 points is obtained.
